# Quantitative Evaluation of *in Vivo* Target Efficacy of Anti-tumor Agents *via* an Immunofluorescence and EdU Labeling Strategy

**DOI:** 10.3389/fphar.2018.00812

**Published:** 2018-07-25

**Authors:** Yujun He, Jin Wen, Qinghua Cui, Fangfang Lai, Dali Yin, Huaqing Cui

**Affiliations:** ^1^State Key Laboratory of Bioactive Substance and Function of Natural Medicines, Institute of Materia Medica, Chinese Academy of Medical Sciences and Peking Union Medical College, Beijing, China; ^2^Department of Urology, Peking Union Medical College Hospital, Beijing, China; ^3^College of Pharmacy, Shandong University of Traditional Chinese Medicine, Jinan, China

**Keywords:** anti-tumor drug, *in vivo*, target efficacy, quantitative analysis, EdU labeling, immunofluorescence

## Abstract

Current methods used to evaluate *in vivo* target efficacy of selected compound include western blot to semi-quantitatively analyze protein expression. However, problems arise as it is difficult to compare *in vivo* target efficacy of anti-tumor agents with the same mode of action. It is therefore desirable to develop a protocol that can quantitatively display *in vivo* target efficacy while also providing other useful information. In this study EdU labeling was used to mark out the proliferating area. The tumor tissue was accordingly divided into proliferating and non-proliferating areas. Fifteen tumor related proteins were stained by immunofluorescence and were found to express in either the proliferating or non-proliferating areas. This allows the quantitative analysis of protein expressions within the precise area. With simple image analysis, our method gave precise percent changes of protein expression and cell proliferation between the drugs treated group and the control group. Additional information, such as, the status of protein expression can also be obtained. This method exhibits high sensitivity, and provides a quantitative approach for *in vivo* evaluation of target efficacy.

## Introduction

The discovery of small molecule anti-tumor clinical candidates relies upon several critical steps. One of these steps is the evaluation of the *in vivo* activity of compounds against human xenograft tumor in mouse models. This important step typically takes place once a discovery campaign have identified compounds that display potent *in vitro* target inhibitory activities (Burger et al., 2011; Luconi and Mannelli, 2012; Falcon et al., 2013; Simeoni et al., 2013; Zhao et al., 2016; An et al., 2018). Upon administering the compounds to animals, the expression levels of drug related proteins can change. The current method to observe these changes relies upon western blot to semi-quantitatively analyze the expressions of proteins of interest (Luconi and Mannelli, 2012; An et al., 2018). In addition, the immunohistochemistry or immunofluorescence is also used to symbolic display the expression changes of protein of interested without any quantitative standard. Typically, after dosing with compounds, the mice were euthanized and the tumors were excised. The tumor is then ground to extract the proteome present within the tumor, and the expressions of proteins were analyzed *via* western blot, compared among different experimental groups. This method is generally reliable and widely accepted by researchers, but it does suffer from sensitivity and quantity issues. This is most notable when the inhibitors used do not have strong *in vivo* activity (Falcon et al., 2013). Particularly, this method is difficult to compare the *in vivo* target efficacy of different compounds with similar mode of action, while this information is important for the selection of candidate compounds for further development.

An effective strategy to combat this issue without making any changes to compounds’ administration, is to analyze the expression of target proteins within a precise sectional area of tumor tissue instead of the whole tumor. This precise area in tumor tissue can play the role of quantitative standard as the control protein in traditional western blot analysis, and should allow the accurate comparison of target proteins expression among different samples. Ideally, the selected area of tumor tissue should be representative and unbiased as well accurately reflecting the biological processes of drug administration. Generally, cancer researchers divide solid tumors into proliferating area and non-proliferating area (Hanahan and Weinberg, 2000, 2011; Cui et al., 2010; Murar and Vaidya, 2015). In this study, we will test the possibility to evaluate compounds’ *in vivo* target efficacy in proliferating/non-proliferating area instead of the whole tumor tissue.

Various methods have been developed to robustly label proliferating cells in tumor tissues. Unnatural nucleosides, such as, 5-bromo-2′-deoxyuridine (BrdU) (Gratzner, 1982; Wang et al., 2018; Yan et al., 2018), 5-ethynyl-2′-deoxyuridine (EdU) (Salic and Mitchison, 2008) and 5-vinyl-2′-deoxyuridine (VdU) (Rieder and Luedtke, 2014), are used to initially incorporate into replicated DNA, and these unnatural nucleosides can be subsequently labeled through immunostaining or bioorthogonal reactions. Among them, EdU labeling is simple and fast, and EdU labeling has been widely used to recognize proliferating cells in cultured cells and tissues (Limsirichaikul et al., 2009; Lentz et al., 2010; Neef and Luedtke, 2011; Neef et al., 2012). EdU labeling requires mild conditions, and does not cause any damage to protein epitopes, which allows it to be used in a concurrent fashion with protein immunostaining.

The measurement of protein expression depends heavily on antibody based methods, such as, western blot, flow cytometry, immunohistochemistry, and immunofluorescence. In comparison to western blot analysis, the other three methods have the capability to determine the percentage of cells that expresses the protein (Wijsman et al., 2007). Flow cytometry is often used to analyze cultured cells with immunohistochemistry and immunofluorescence particularly suited to the analysis of the expression status of cells in tissues (Pusztai et al., 2006; Wijsman et al., 2007).

In this study, we aimed to develop a quantitative method to evaluate *in vivo* target efficacy. Instead of treating the tumor as a whole entity, the tumor tissue was divided into proliferating and non-proliferating areas. This allows the comparison of protein expressions within a precise and consistent area among various samples.

## Materials and Methods

### Materials

Chemicals and solvents were purchased from Admas reagents, J&K chemical, Shanghai Macklin biochem tech, Sigma-Aldrich and RIBOBIO Guangzhou. The antibodies were purchased from ZENBIO Chengdu, Cell Signaling Technology Inc. and ZSGB-BIO Beijing. Click-iT^TM^ cell reaction buffer kit (C10269) was obtained from Invitrogen. Compounds (BKM120, PX-478) were purchased from Selleck. DAB kit (ZLI-9017) was obtained from ZSGB-BIO Beijing.

Human lung carcinoma cell line H460, human breast cancer cell line MCF-7 and mouse colon adenocarcinoma cell line MC38 were obtained from the Institute of Medical Sciences, Peking Union Medical College (Beijing, China) and Obio Technology (Shanghai, China). Cells were maintained in the medium of DMEM or RPMI 1640 (Hyclone) supplemented with 10% heat-inactivated FBS (Hyclone) at 37°C in a humidified incubator containing 5% CO_2_.

### Drug Administration to Tumor Xenograft Mice

Adult female C57BL/6 mice and nude mice (8–10 weeks of age, 18–22 g) were purchased from Vital River Laboratory Animal Technology Co., Ltd. (Beijing, China). All animal experiments were conducted in compliance with the Care and Use of Laboratory Animals with the approval of Peking Union Medical College and Chinese Academy of Medical Sciences’ Animal Studies Committee. H460, MCF-7 and MC38 cells (1 × 10^7^ cells/mL) were cultured, harvested and re-suspended in saline. 100 μl each cell suspension was subcutaneously injected into the right flank of each mouse. Human MCF-7 and H460 xenograft tumors were planted on nude mice, mouse MC38 xenograft tumors were planted on C57BL/6 mice.

When the tumor volume reached approximately 80∼100 mm^3^, the mice were ready for dosing studies. For experiment 1: mice with MCF-7 xenograft tumors were treated with different doses (5, 12.5, 25, 50, or 100 mg/kg) of EdU. For experiment 2: mice with MCF-7, H460, and MC38 xenograft tumor were treated with EdU (50 mg/kg) for various periods (3, 6, 12, 24, and 48 h) before being sacrificed. For experiment 3, mice with MCF-7 xenograft tumor were intraperitoneally injected with EdU at a dose of 50 mg/kg body weight and were sacrificed after 8 h. For experiment 4, mice with H460 xenograft tumor were orally treated with BKM120 (45 mg/kg/day) for 5 days, and were sacrificed after 1 h of the last administration of BKM120. For experiment 5, mice with H460 xenograft tumor were intraperitoneally injected with PX-478 at a dose of 100 mg/kg/day for 5 days and were sacrificed after 1 h following the last injection. During experiment 4 and 5, EdU was administrated at a dose of 50 mg/kg 8 h before sacrifice.

### Immunohistochemistry or Immunofluorescence

The tumors were excised and fixed in 4% polyoxymethylene and preserved in 10% neutral buffered formalin. Fixed tumor xenograft tissues were embedded in paraffin and cut into 5 μm thick slices. Before analysis, samples were cleaned of paraffin and rehydrated as follows: heating 30 min at 65°C, washing with xylene, ethanol, ethanol/water mixtures, and finally washing with distilled water. The tissues were treated with 1% Triton X-100 for 15 min at room temperature, and then incubated with 3% H_2_O_2_ in ddH_2_O for 20 min at room temperature. The slices were immersed in citrate buffer (pH 6.0, prepared from sodium citrate dehydrate and citric acid monohydrate) for 30 min at 100°C by microwave for antigen retrieval. The slices were then cooled down to room temperature over time. The slices were immersed in 5% BSA in PBS for 1 h at 37°C before incubation with primary antibody.

60 μl primary antibody was diluted in PBS containing 1% BSA (Supporting Information S1), the antibody solution was added to each slice and incubated overnight at 4°C. The slices were warmed to room temperature naturally, and were washed three times with PBS buffer. 100 μl peroxidase-conjugated or FITC- conjugated secondary antibody were added to the slices, and were incubated for 30 min at 37°C. For peroxidase visualization, the sections were incubated with freshly prepared DAB solution. The reaction was stopped by washing in running water when a uniform brown color first becomes visible on the slices. The slices were then observed under fluorescent microscope.

### EdU Labeling

The slices were incubated with 60 μl freshly diluted Apollo^®^567-N_3_ or Apollo^®^488-N_3_ work solution (3 μM fluorescent-azide, 1 mM CuSO_4_, 10 mM sodium ascorbate in PBS buffer; Invitrogen) for 30 min at 37°C in the dark (Miao et al., 2015). The slices were washed with PBS buffer for three times and with methanol for one time. The slices were observed under fluorescent microscope.

### Image Analysis

The software Image-Pro Plus (Media Cybernetics, United States) was used to analyze the images. The images were firstly divided into the proliferating and non-proliferating areas *via* EdU labeling. To analyze tumor proliferation, we randomly labeled three circles within the EdU area of tumor tissue. The numbers of positive proliferating cells (red fluorescence) were counted within these circles, and the average number of proliferating cells per area unit was calculated and compared among different samples.

To analyze protein expression, we randomly labeled three circles within the EdU area if the proteins were expressed in proliferating area. On the contrary, for the proteins expressed in non-proliferating area, we randomly labeled three circles within the non-EdU labeled area. The numbers of protein expressed cells (green fluorescence) were counted within these circles, and the average number of protein expressed cells per unit area was calculated and compared among different samples.

## Results and Discussion

### Marking Out the Proliferating Area in Tumor Tissue by EdU Labeling

**FIGURE 1:**
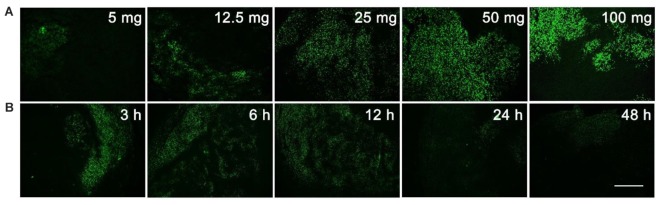
Screening for the optimal dosage and administration time of EdU labeling. **(A)** Various dosage (5, 12.5, 25, 50, or 100 mg/kg) of EdU was intraperitoneally injected to mice for 12 h. **(B)** 50 mg/kg EdU was intraperitoneally injected to mice for various times (3, 6, 12, 24, and 48 h). Scale bar indicates 2 mm.

The unnatural nucleoside EdU has been extensively used to label proliferating cells in tissue (Vega and Peterson, 2005; Chehrehasa et al., 2009; Miao et al., 2015), but we re-visited this experiment to determine the optimal dosage and administration time for EdU labeling. Mice with MCF-7 xenograft tumor were used in this study. Firstly, mice were intraperitoneally injected with various dosages (5, 12.5, 25, 50, or 100 mg/kg) of EdU, and were sacrificed 12 h after injection. EdU was labeled with Apollo^®^488-N_3_ probes through a click chemistry reaction. From **Figure 1A**, we can see that a relatively weak fluorescence can be observed in the dosage group of 5 mg/kg EdU. The fluorescent intensity increased in a dose dependent manner and reached peak level intensity at 50 mg/kg EdU. After confirming the optimal dose was 50 mg/kg, we then treated the xenograft tumor mouse model at this dose over various times (3, 6, 12, 24, and 48 h). The results in **Figure 1B** showed that EdU can be detected among a small area of tumor tissue in the 3 h group, and the fluorescent area reached peak levels within the 6 and 12 h groups. We also observed that the fluorescent intensity decreased in both the 24 and 48 h group, which is possibly due to cell division after DNA replication or possibly a second round of DNA replication. Thus, we chose the EdU injection time as 8 h, which is between 6 and 12 h (Supporting Information S2). Mice with MC38 and H460 xenograft tumors were also used to screen for the optimal EdU labeling conditions in tumor tissues (Supporting Information S2) and observed the same optimal labeling conditions in these two models. In summary, we concluded that the conditions for EdU labeling xenograft tumor mouse model: the mouse was intraperitoneally injected with EdU (50 mg/kg) for 8 h.

### The Combination of EdU Labeling and Immunofluorescence

**FIGURE 2:**
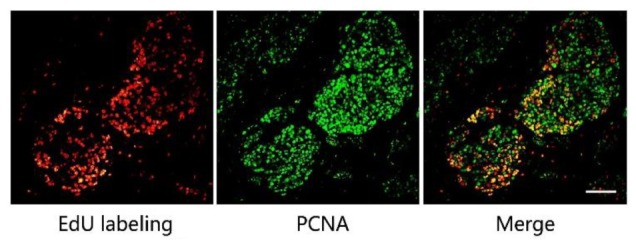
The combination of EdU labeling and immunofluorescence of PCNA. Red fluorescence shows the labeling of EdU with Apollo^®^567-N_3_ and green fluorescence represents the immunofluorescence of PCNA with FITC-Conjugated Mouse anti-Goat IgG H&L. The images of EdU labeling and PCNA immunofluorescence were superimposed. Scale bar indicates 2 mm.

We then optimized the procedure to combine antibody immunostaining with EdU labeling to achieve high quality merged images. Mice with MCF-7 xenograft tumors were used in this study and the proliferating cell nuclear antigen (PCNA) was chosen as the model protein. We firstly performed EdU labeling in tumor slices, followed PCNA immunofluorescence. The images of EdU labeling and the immunofluorescence were subsequently superimposed for analysis. The result shows that the EdU labeled proliferating areas overlapped well with PCNA expression (**Figure 2**). In addition, we also performed the immunofluorescence first and followed EdU labeling, and observed no difference in the results obtained. However, when immunochemistry was used to replace the immunofluorescence, we found that fluorescence of EdU labeling is acutely interrupted by the immunochemistry staining (Supporting Information S3).

PCNA has been used as the biomarker to detect cell proliferation (Miyachi et al., 1978; Muskhelishvili et al., 2003), and EdU was used to label the proliferating cells. Thus, the positive area of PCNA and the area of EdU labeling should be overlapped in the same tumor tissue as shown in **Figure 2**. This also supports the fact that the EdU labeling area represents the proliferating area. In this study, the mild condition required for EdU labeling allows for the concurrent use of antibody immunostaining, with either immunofluorescence or EdU labeling being conducted first. However, as we mentioned above, immunohistochemistry is not suitable for combination with EdU labeling. In the immunohistochemistry experiments, reaction between horseradish peroxidase and its substrate, 3, 3′-diaminobenzidine (DAB), formed a brown precipitate on the slide, which directly affected the performance of the EdU labeling.

### The Correlation Between of the Expressions Areas of Cancer Related Proteins and the EdU Labeled Proliferating Area

We then investigated the correlation between the expression areas of cancer related proteins and the EdU labeled proliferating area. We chose mice with MCF-7 xenograft tumors for the study. We selected 15 reported cancer related proteins (**Table 1**) to be investigated.

**Table 1:** List of selected 15 cancer related proteins.

Protein name	Abbreviation	Protein name	Abbreviation
Apoptosis inducing factor	AIF	Cyclin-dependent kinase-1	CDK 1
Protein kinase B	AKT	DNA-dependent protein kinase catalytic subunit	DNA-PKcs
Aurora Kinase A	Aurora A	E2F	E2F
Epidermal growth factor receptor	EGFR	Focal Adhesion Kinase	FAK
Glycogen synthase kinase 3 beta	GSK 3β	Hypoxia inducible factor-1 alpha	HIF 1α
Lupus Ku autoantigen protein p80	KU 80	Phospho-Cyclin-dependent kinase-1	p-CDK 1
M2-type pyruvate kinase	PKM 2	Structural maintenance of chromosomes protein 1A	SMC1A
Signal transducer and activator of transcription 3	Stat3		

**FIGURE 3:**
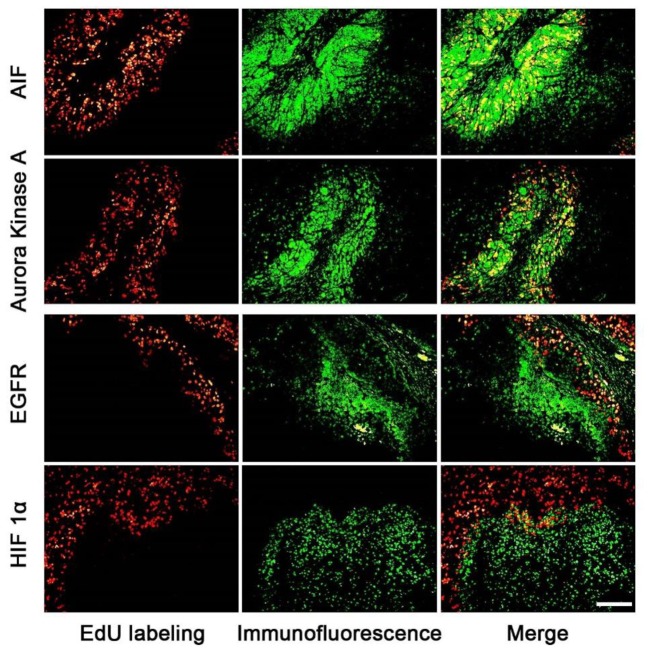
The correlation of the expression area of cancer related proteins and the proliferating area in tumor tissues. The images of cancer related proteins (green) and the proliferating area (red) were superimposed to observe the correlation of them. The proteins of AIF and Aurora kinase A mainly expressed in the proliferating areas, but EGFR and HIF proteins mainly expressed in the non-proliferating area. Scale bar indicates 2 mm.

The expression areas of all 15 proteins were analyzed in the tumor slice (**Figure 3** and supporting Information S4). Interestingly, all 15 proteins were expressed in either the proliferating (**Figure 3**: AIF and Aurora Kinase A) or non-proliferating (**Figure 3**: HIF 1α and EGFR) area. **Figure 3** listed four proteins’ expression images, the expression images of the rest proteins are shown in the supporting information S4. For example, the expression areas of AIF and Aurora Kinase A overlapped well with the EdU labeled proliferating areas whereas EGFR and HIF 1α only expressed in the non-proliferating area of tumor tissues. We observed that the numbers of proteins found in the proliferating area (13 proteins) far exceeds that found in the non-proliferating area (2 proteins).

Development of tumors is a complex process involving multiple genes. Both the proliferating and non-proliferating areas of tumor tissues are important in the development of cancer (Cantor and Sabatini, 2012). In this study, 15 well studied cancer related proteins were chosen to explore the correlation between of the expressions areas of them and the EdU labeled proliferating area. A publication list of these 15 cancer related proteins was included in the Supporting Information S4 and Supplementary Table S2. Many of these cancer related proteins are validated anti-tumor drug targets, and specific inhibitors or antibodies against them are commercial available on the market.

Our results showed that the cancer related proteins, such as, AIF and Aurora Kinase A, were expressed in the proliferating area of tumor tissues. AIF is caspase-independent apoptosis factor, which plays an important role in embryonic development, which maintains cell survival and induces cell apoptosis (Churbanova and Sevrioukova, 2008) while Aurora Kinase A participates in the whole progress of mitosis (Ikezoe et al., 2007; Lassmann et al., 2007; Baba et al., 2009). On the contrary, two cancer related proteins, such as, EGFR and HIF 1α, were found to be mainly expressed in the non-proliferating area of the tumor tissue. EGFR involves the growth of vessels (Vara et al., 2004; Martini et al., 2014; Hahne et al., 2017) and HIF 1α protein is related to tissue hypoxia. The expression areas of cancer related proteins are related to the biological functions.

The expression of cancer related proteins are highly correlated to the proliferating/non-proliferating area of tumor tissue, therefore the EdU labeled proliferating/non-proliferating areas can be used as the quantitative standard to evaluate the target expressions in tumor tissue. The assignment of a precise area in tumor tissues to observe the change of protein expression has two important implications. Firstly, the assignment of a partial area instead of the whole tumor can amplify expression changes and therefore increase the sensitivity of the measurement and assessment. Secondly, the assignment of a precise area allows the comparison of the expression status of proteins among different samples. A precise area is consistent in different samples, which plays as the control standard to allow the accurate analysis.

### Quantitative Evaluation of *in Vivo* Target Efficacy of Anti-tumor Agents Within EdU/non-EdU Area

**FIGURE 4:**
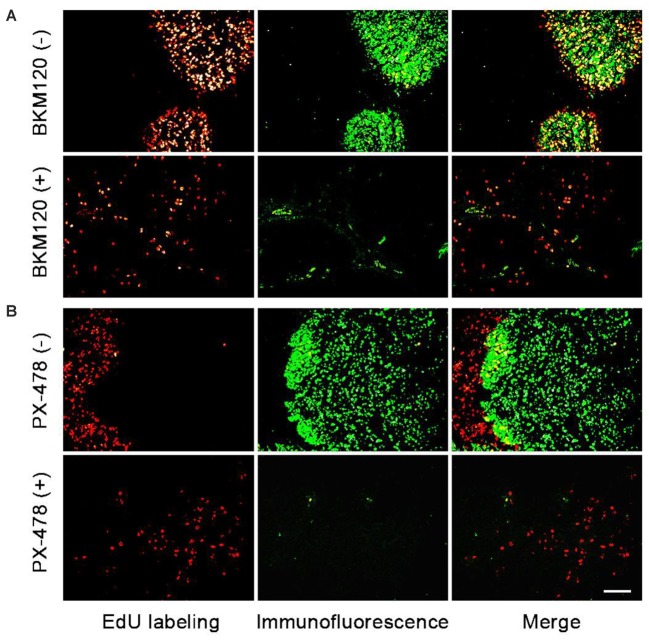
Evaluation of *in vivo* target efficacy of BKM120 and PX-478. The green fluorescence show the expression of the target proteins Akt and HIF 1α. The red fluorescence show the EdU labeling. **(A)** Mice with MCF-7 xenograft tumor were treated with BKM120, phosphorylated Akt was examined. **(B)** Mice with H460 xenograft tumor were treated with PX-478, HIF 1α was detected. Scale bar indicates 2 mm.

Next, we chose two pairs of target/inhibitor to evaluate their *in vivo* target efficacy: Akt protein mainly expressed in the proliferating area, and HIF 1α mainly expressed in the non-proliferating area. For our first example, BKM120 is a reported orally available PI3K inhibitor (Burger et al., 2011). The inhibition of PI3K by BKM120 will affect the phosphorylation of Akt protein. Mice with MCF-7 xenograft tumor were treated with BKM120 (45 mg/kg/day) for 5 days. EdU was injected 8 h before sacrifice. **Figure 4A** shows that the phosphorylated Akt protein mainly expressed in the proliferating area of tumor tissue and the expression region of phosphorylated Akt overlapped with EdU labeling area. After treatment with BKM120, the expression of phosphorylated Akt remained in the proliferating area. However, the number of cells that expressed phosphorylated Akt protein dramatically reduced. In addition, the intensity of proliferating cells in the proliferating area was much lower in the BKM120 treated group compared to the control group (**Figure 4A**).

For the second case, PX-478 is a highly potent and selective HIF 1α inhibitor (Welsh et al., 2004). Mice with H460 xenograft tumor were treated with PX-478 (100 mg/kg/day) for 5 days. EdU was injected 8 h before the sacrifice. **Figure 4B** showed that HIF 1α mainly expressed in the non-proliferating area of tumor tissue. However, after PX-478 treatment, the expression of HIF 1α is marginally present in the edge of non-proliferating areas. In addition, the intensity of proliferating cells also decreased significantly in the proliferating area in PX-478 treated tumor.

**FIGURE 5:**
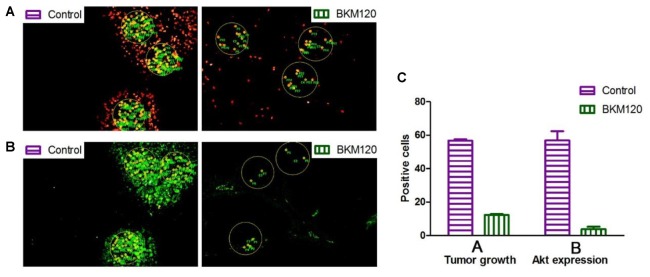
Image analysis to access cell proliferation and protein expression. **(A)** The proliferating cells were labeled with EdU in both control and BKM120 treated groups. **(B)** The expression of phosphorylated Akt were stained by immunofluorescence in both control and BKM120 treated groups. **(C)** Quantitative analysis of positive cells per area unit regarding of the proliferating cells and the expression of phosphorylated Akt in both control and BKM120 treated groups.

Next, we analyzed the images to compare inhibition rates of both protein expression and tumor proliferation among different groups. The image software Image-Pro Plus was used to analyze the images. In **Figure 5A**, we separately labeled three circles in the EdU area of control group and BKM120 treated groups. The positive cells per area unit were calculated for each sample. The results showed that BKM120 administration reduced 78.4% cell proliferation compared to the control group (**Figure 5C**). In addition, the expression stage of Akt protein were also evaluated between the control and the BKM120 treated groups (**Figure 5B**). A 93.4% Akt expression was reduced after BKM120 treatment. For the case of HIF 1α target, after the image analysis, we found that 98.6% HIF 1α protein expression was inhibited by PX748 administration, and the tumor growth was slow down for 80.3% (Supplementary Figure S5).

Akt is a downstream protein of PI3K, and it is well known that PI3K can promote cell proliferation and metabolism by regulating phosphorylation of its downstream substrates (Cristofano et al., 1998; Suzuki et al., 1998). When PI3K was inhibited by BKM120, the phosphorylation of Akt was also inhibited. HIF protein is related to tissue hypoxia, increased HIF-1α expression in response to hypoxia can increase growth factor expression to induce angiogenesis (Pennacchietti et al., 2003; Carroll and Ashcroft, 2006). The administration of mice with inhibitors of BKM120 or PX-478 can inhibit the growth of tumors. We showed that the quantitative evaluation of *in vivo* target efficacy of BKM120 and PX-478. Firstly, a clear change in the target protein expression before and after the treatment can be observed, which exhibits the *in vivo* target efficacy of the anti-tumor agents. After image analysis, a precise percent change can be obtained between drug treated and control groups. Secondly, the intensity of the proliferating cells in the proliferating area can be calculated, which correlates to the growth status of the tumor. Lastly, compared to semi-quantitative western blot analysis, our method is more sensitive and can be used to evaluate of *in vivo* target efficacy of various inhibitors. Thus, this method can deliver more therapeutically useful information. In particular, the visual analysis can be used in combination with various imaging analysis platform, such as, high content screening, to present more useful information for drug discovery projects (Falcon et al., 2013; Lai et al., 2017).

## Conclusion

In this study, we used EdU labeling to mark the proliferating area of tumor tissue. The high correlation between EdU labeled proliferating/non-proliferating areas and the expression areas of cancer related proteins suggest that EdU labeling can be used as the control standard for the assessment of protein expression. The images of immunofluorescence and EdU labeling were analyzed to give the precise percent changes of protein expression and tumor proliferation between control and drug treated groups. This allows the direct comparison of the *in vivo* efficacy of anti-tumor agents with similar mode of actions. Taken together, the combination of immunofluorescence and EdU labeling can be used to quantitatively evaluate *in vivo* target efficacy of anti-tumor agent while also providing other information.

## Author Contributions

YH, JW, and QC conducted the experiments and analyzed the data. HC, FL, and DY designed the experiments. YH and HC wrote the article.

## Conflict of Interest Statement

The authors declare that the research was conducted in the absence of any commercial or financial relationships that could be construed as a potential conflict of interest.
