# Supplementary material for: Quantitative Evaluation of in Vivo Target Efficacy of Anti-tumor Agents via an Immunofluorescence and EdU Labeling Strategy
Source: Front Pharmacol. 2018 Jul 25;9:812. doi: 10.3389/fphar.2018.00812 (PMC6077270; doi:10.3389/fphar.2018.00812)
Supplement: Supplementary file 1 [file Data_Sheet_1.PDF]

Supplementary Material

Quantitative evaluation of *in vivo* target efficacy of anti-tumor agents *via* an immunofluorescence and EdU labelling strategy

Yujun He[1, †], Jin Wen[2, †], Qinghua Cui[3], Fangfang Lai[1*], Dali Yin[1], Huaqing Cui[1*]

[1]State Key Laboratory of Bioactive Substances and Function of Natural Medicine, Institute of Materia Medica, Peking Union Medical College and Chinese Academy of Medical Sciences, Beijing, 100050, China

[2]Department of Urology, Peking Union Medical College Hospital, Beijing, 100730, China

[3]College of Pharmacy, Shandong University of Traditional Chinese Medicine, Jinan, 250355, China

*** Correspondence:**
Huaqing Cui
hcui@imm.ac.cn

Fangfang Lai
laifangfang@imm.ac.cn

1 Supporting Information S1

The information of used antibodies was listed in table S1.

Table S1: The information of antibodies were listed.

**Antibodies**	**Catalog #**	**Dilution ratio[a]**	**Company**
AIF	201149-8H1	1: 200	ZENBIO Chengdu
Akt	342529	1: 50	
Aurora Kinase A	200525	1: 50	
CDK 1	500220	1: 100	
DNA-PKcs	200618-6d1	1: 100	
E2F	201080	1: 100	
FAK	200899	1: 200	
GSK 3β	200494-2E6	1: 200	
HIF 1α	380825	1: 50	
Ku 80	201004	1: 100	
p-CDK 1 (Y15)	530016	1: 50	
PKM 2	200667	1: 200	
SMC1A(N-term)	200256	1: 100	
Stat 3(1E3)	250208	1: 200	
EGFR(D38B1)	4267S	1: 200	Cell Signaling Technology
PCNA	ZM-0213	1: 100	ZSGB-BIO Beijing

FITC-Conjugated Mouse anti-Goat IgG H&L	ZF-0312	1: 100	
FITC-Conjugated Rabbit anti-Goat IgG H&L	ZF-0314	1: 100	
Peroxidase-Conjugated Mouse anti-Goat IgG H&L	ZB-2305	1: 100	
Peroxidase-Conjugated Rabbit anti-Goat IgG H&L	ZB-2306	1: 100	

[a]: diluted in 1% BSA

2 Supporting Information S2

Administrated time of EdU in different tumor models was studied. As illustrated in Fig S1, after 6-8 hours of EdU injection, the labelled area reached to the peak level. Thus, the optimal administration time of EdU was 8h in H460 and MC38 tumor model.

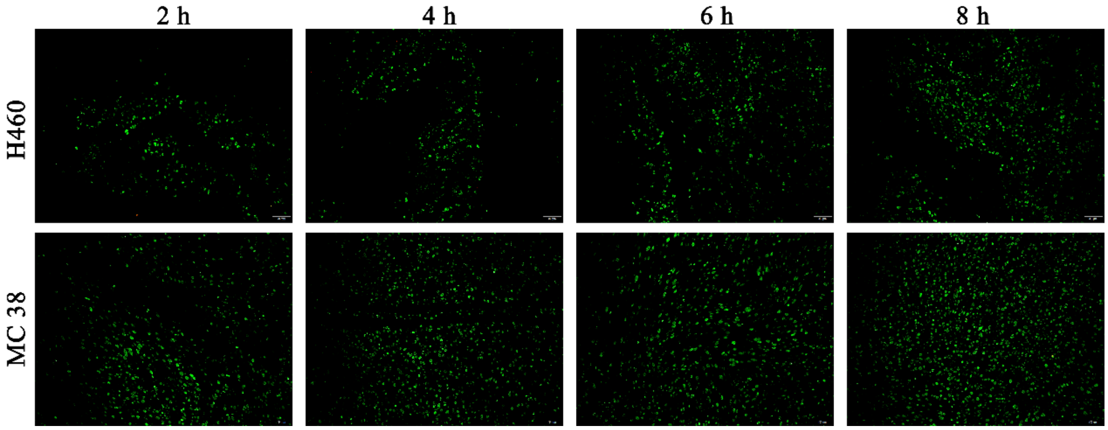

Figure S1: Effect of different administration time for EdU labelling. EdU was stained with FITC-azide (Apollo®488-N_3).

3 Supporting Information S3

Staining reaction time of DAB have a significant interruption on EdU labelling in the same tissue section.

In IHC assay of PCNA, 10 seconds DAB staining time is too long and eventually the precipitation covers the EdU labelling, while 5 seconds DAB staining time is feasible to observe the EdU labelling.

In generally, we do not recommend the application of immunohistochemistry to be used with the combination of EdU labelling.

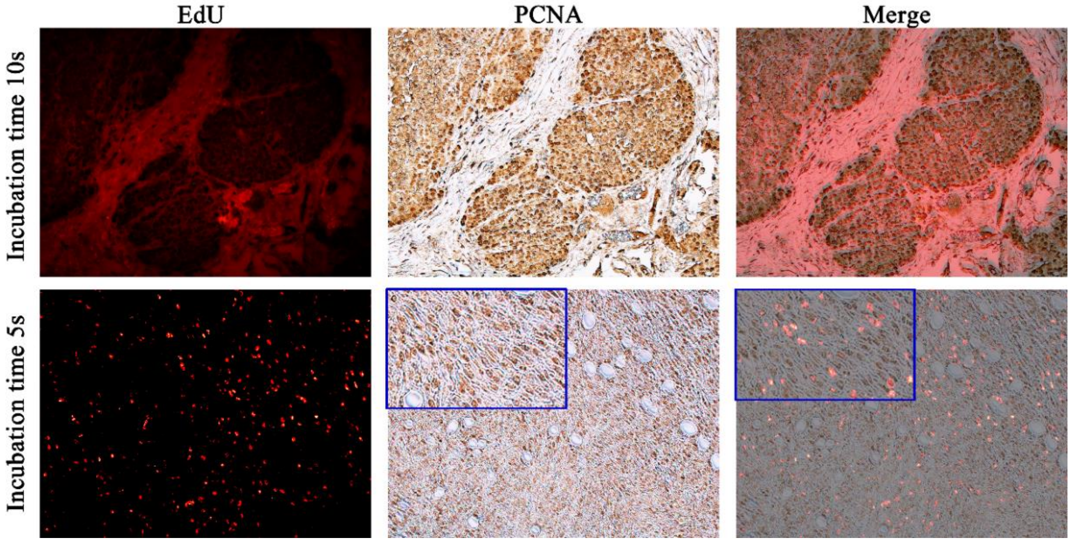

Figure S2: Establish of IHC-EdU based multiplexed tissue imaging method. Detection of EdU was performed by Apollo®567-N_3 (Red). PCNA was stained with DAB (Brown).

4 Supporting Information S4

15 proteins were screened the correlation between the expression area and EdU labelled proliferating/non-proliferating area.

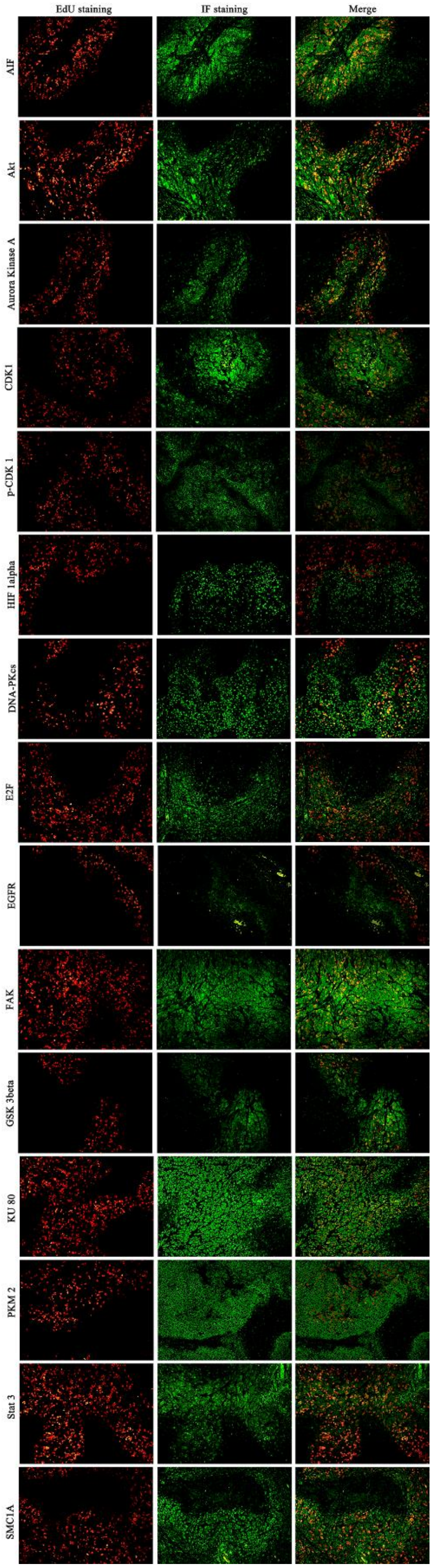

Figure S3: The correlation of the expression area of cancer related proteins and the proliferating area in tumour tissues. Proteins were stained with FITC-Conjugated anti-Goat IgG H&L (Green) and EdU reacted with Apollo®567-N3 (Red).

Table S2: Publication list of selected 15 cancer related proteins

Protein Name	Selected Publications
Apoptosis inducing factor (AIF)	AIFsh, a novel apoptosis-inducing factor (AIF) pro-apoptotic isoform with potential pathological relevance in human cancer. J Biol Chem. 2006; 281(10):6413-27.<br><br>Apoptosis-Inducing Factor and Colon Cancer. J Surg Res. 2009; 151(1):163-70.<br><br>Essential role of the mitochondrial apoptosis-inducing factor in programmed cell death. Nature. 2001; 410(6828):549-54.
Cyclin-dependent kinase-1 (CDK 1)	Cyclin-Dependent Kinase 1 Gene Expression Is Associated with Poor Prognosis in Gastric Carcinoma. Clin Cancer Res. 2003; 9(15):5693-8.<br><br>Cyclin-Dependent Kinase Pathways As Targets for Cancer Treatment. J Clin Oncol. 2006; 24(11):1770-83.<br><br>Targeting cyclin-dependent kinase 1 (CDK1) but not CDK4/6 or CDK2 is selectively lethal to MYC-dependent human breast cancer cells. BMC Cancer 2014, 14:32
Protein kinase B (AKT)	Current overview of the role of Akt in cancer studies via applied immunohistochemistry. Ann Diagn Pathol. 2008; 12(2):153-60.<br><br>Essential role of AKT in tumor cells addicted to FGFR. Anticancer Drugs. 2014; 25(2):183-8.<br><br>The Akt pathway in human breast cancer: a tissue-array-based analysis. Mod Pathol. 2006; 19(2):238-45.
DNA-dependent protein kinase catalytic subunit (DNA-PKcs)	DNA-PKcs Expression in Esophageal Cancer as a Predictor for Chemoradiation Therapeutic Sensitivity. Ann Surg Oncol. 2002; 9(10):1017-22.<br><br>Mutual regulation between DNA-PKcs and snail1 leads to increased genomic instability and aggressive tumor characteristics. Cell Death Dis. 2013; 4:e517.<br><br>Role of DNA-dependent protein kinase catalytic subunit in cancer development and treatment. Transl Cancer Res. 2012; 1(1): 22–34.

Aurora Kinase A (Aurora A)	Aurora kinase A and B as new treatment targets in aromatase inhibitor-resistant breast cancer cells. Breast Cancer Res Treat. 2015; 149(3): 715-26.
E2F	Emerging roles of E2Fs in cancer: an exit from cell cycle control. Nat Rev Cancer. 2009; 9(11):785-97.<br><br>Disruption of Rb/E2F pathway results in increased cyclooxygenase-2 expression and activity in prostate epithelial cells. Cancer Res. 2005; 65(9):3633-42.
Epidermal growth factor receptor (EGFR)	Epidermal growth factor receptor mutations in lung cancer. Nat Rev Cancer. 2007; 7(3):169-81.<br><br>Rational, biologically based treatment of EGFR-mutant non-small-cell lung cancer. Nat Rev Cancer. 2010; 10(11):760-74.<br><br>Mutations of the Epidermal Growth Factor Receptor Gene in Lung Cancer: Biological and Clinical Implications. Cancer Res. 2004; 64(24):8919-23.
Focal Adhesion Kinase (FAK)	Focal adhesion kinase and cancer. Histol Histopathol. 2009; 24: 503-510.<br><br>Signal transduction by focal adhesion kinase in cancer. Cancer Metastasis Rev.2009; 28:35–49.<br><br>The role of focal-adhesion kinase in cancer - a new therapeutic opportunity. Nat Rev Cancer. 2005; 5(7):505-15.
Glycogen synthase kinase 3 beta (GSK 3β)	Glycogen synthase kinase 3b (GSK3b) in tumorigenesis and cancer chemotherapy. Cancer Letters, 2009; 273:194 – 200.<br><br>Glycogen synthase kinase 3 beta: can it be a target for oral cancer. Mol Cancer. 2010; 9:144.
Hypoxia inducible factor-1 alpha (HIF 1α)	Defining the role of hypoxia-inducible factor 1 in cancer biology and therapeutics. Oncogene, 2010; 29, 625–634.<br><br>Hypoxia-inducible factor 1 alpha in high-risk breast cancer: an independent prognostic parameter? Breast Cancer Res. 2004; 6(3):R191-8.<br><br>Inhibiting Hypoxia-Inducible Factor 1 for Cancer Therapy. Mol Cancer Res 2006; 4(9), 601-605.

Lupus Ku autoantigen protein p80 (KU 80)	DNA repair protein Ku80 suppresses chromosomal aberrations and malignant transformation. Nature. 2000; 404(6777):510-4.<br><br>Synergistic Role of Ku80 and Poly(ADP-ribose) Polymerase in Suppressing Chromosomal Aberrations and Liver Cancer Formation. Cancer Res. 2002; 62(23):6990-6.<br><br>The biology of Ku and its potential oncogenic role in cancer. Biochimica et Biophysica Acta, 2006; 1765: 223 – 234
Phospho-Cyclin-dependent kinase-1 (p-CDK 1)	Cell cycle, CDKs and cancer: a changing paradigm. Nat Rev Cancer. 2009; 9(3):153-66<br><br>The cell cycle: a review of regulation, deregulation and therapeutic targets in cancer. Cell Prolif. 2003, 36, 131–149
M2-type pyruvate kinase (PKM 2)	Emerging roles of PKM2 in cell metabolism and cancer progression. Trends Endocrinol Metab. 2012; 23(11):560-6.<br><br>Shikonin and its analogs inhibit cancer cell glycolysis by targeting tumor pyruvate kinase-M2. Oncogene.2011, 30, 4297–4306.<br><br>Tyrosine Phosphorylation Inhibits PKM2 to Promote the Warburg Effect and Tumor Growth. Sci Signal. 2009; 2(97):ra73.
Structural maintenance of chromosomes protein 1A (SMC1A)	Low SMC1A protein expression predicts poor survival in acute myeloid leukemia. Oncol Rep. 2010; 24(1):47-56.<br><br>Role of SMC1A overexpression as a predictor of poor prognosis in late stage colorectal cancer. BMC Cancer. 2015; 15:90.<br><br>SMC1A promotes growth and migration of prostate cancer in vitro and in vivo. Int J Oncol. 2016; 49(5):1963-1972.
Signal transducer and activator of transcription 3 (Stat3)	Regulation of the innate and adaptive immune responses by Stat-3 signaling in tumor cells. Nat Med. 2004; 10(1):48-54.<br><br>Signal transducer and activator of transcription 3 (STAT3) activation in prostate cancer: Direct STAT3 inhibition induces apoptosis in prostate cancer lines. Mol Cancer Ther. 2004; 3(1):11-20.<br><br>Signal Transducer and Activator of Transcription 3 Is Required for the Oncogenic Effects of Non – Small-Cell Lung Cancer – Associated Mutations of the Epidermal Growth Factor Receptor. Cancer Res, 2006; 66: (6).

Supporting Information S5

4.1 Akt & BKM120 case

(1), Inhibition of tumor proliferation

	Positive cells	Area Unit	Positive cells /1000 Unit	Mean	SD
Control	38	67886.68	55.976	56.727	0.778
	38	66051.99	57.530		
	39	68813.45	56.675		
BKM120	8	67886.68	11.784	12.276	0.579
	10	77437.12	12.914		
	10	82447.96	12.129		

BKM120 treatment can inhibit the tumor growth：78.4%

(2), Inhibition of Akt expression

	Positive cells	Area Unit	Positive cells / 1000 Unit	Mean	SD
Control	40	66051.99	60.558	56.932	5.456
	33	65144.07	50.657		
	41	68813.45	59.581		
BKM	4	73541.54	5.439	3.738	1.474
	2	69746.50	2.868		

	2	68813.45	2.906		

BKM120 treatment can inhibit the expression of phosphorylated Akt：93.4%

4.2 HIF 1α and PX 478 case

(1), Inhibition of tumor proliferation

	Positive cells	Area Unit	Positive cells/ 1000 Unit	Mean	SD
Control	24	38013.27	63.136	62.356	5.721
	22	35968.09	61.165		
	23	36643.51	62.767		
PX 478	8	71631.45	11.168	12.274	0.961
	10	78426.72	12.751		
	9	69746.50	12.904		

PX-478 treatment can inhibit the tumor growth：80.3%

(2), Inhibition of HIF 1α expression

	Positive cells	Area Unit	Positive cells/ 1000 Unit	Mean	SD
Control	43	54739.11	78.554	76.072	4.849
	38	53912.87	70.484		
	44	55571.63	79.177		
PX 478	2	150673.9	1.327	1.062	0.230

	1	105208.8	0.950		
	1	109858.4	0.910		

PX-478 treatment can inhibit the expression of HIF 1α：98.6%

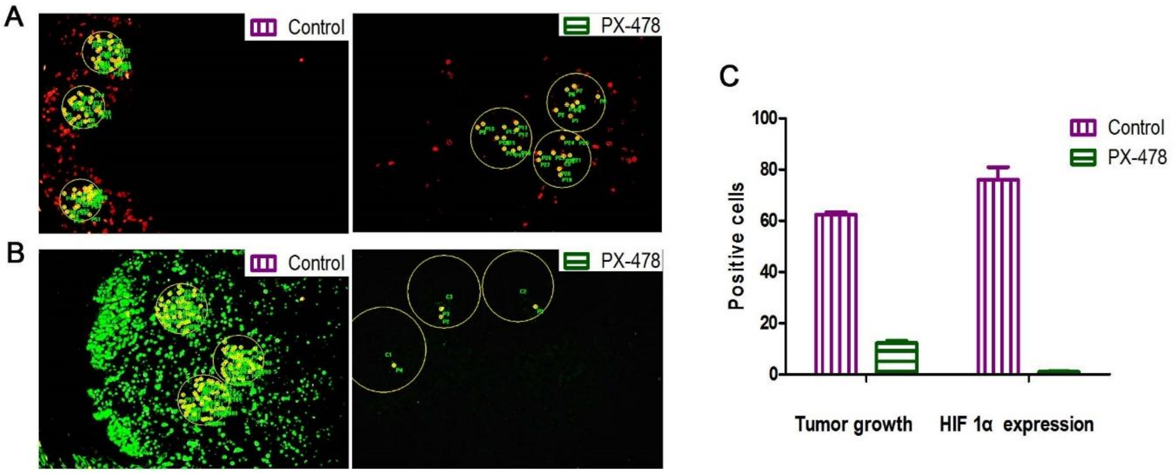

Figure S5: Image analysis to access cell proliferation and protein expression. A, The proliferating cells were labelled with EdU in both control and PX-478 treated groups. B, The expression of HIF 1α were stained by immunofluorescence in both control and PX-478 treated groups. C, Quantitative analysis of positive cells per area unit regarding of the proliferating cells and the expression of HIF 1α in both control and PX-478 treated groups.
